# Structure and elasticity of bush and brush-like models of the endothelial glycocalyx

**DOI:** 10.1038/s41598-017-18577-3

**Published:** 2018-01-10

**Authors:** Aleksei Kabedev, Vladimir Lobaskin

**Affiliations:** School of Physics, University College Dublin, Dublin 4, Ireland

## Abstract

The endothelial glycocalyx (EG), a sugar-rich layer that lines the luminal surface of blood vessels, is an important constituent of the vascular system. Although the chemical composition of the EG is fairly well known, there is no consensus regarding its ultrastructure. While previous experiments probed the properties of the layer at the continuum level, they did not provide sufficient insight into its molecular organisation. In this work, we investigate the EG mechanics using two simple brush and bush-like simulation models, and use these models to describe its molecular structure and elastic response to indentation. We analyse the relationship between the mechanical properties of the EG layer and several molecular parameters, including the filament bending rigidity, grafting density, and the type of ultrastructure . We show that variations in the glycan density determine the elasticity of the EG for small deformations, and that the normal stress may be effectively dampened by the EG layer, preventing the stress from being transferred to the cell membrane. Furthermore, our bush-like model allows us to evaluate the forces and energies required to overcome the mechanical resistance of the EG.

## Introduction

Despite being just several hundred nanometres thick in capillaries^1^, the endothelial glycocalyx (EG) has an enormous impact on a number of vascular functions, playing a major role in sieving and mechanotransduction processes, as well as in the chemical control of the endothelial cell environment. Indeed, it has been shown experimentally that the EG can efficiently filter plasma solutes so that only the smaller molecules can penetrate it^2,3^, and can effectively resist both the pressure exerted by red blood cells (RBCs)^1,4^, and shear stresses exerted by the plasma. While this mechanical resistance of the EG is generally attributed to the high bending rigidity of its constituent molecular fibres, experiments with cultured cells performed by Florian *et al*.^5^, and *in vivo* experiments by Mochizuki^6^, indicated that two cross-linking molecules—heparan sulfate and hyaluronan (constituents of the EG)—may also play an important role in the detection and amplification of flow-induced shear forces. Tarbell and Pahakis have proposed a mechanotransduction hypothesis that assumes that the EG transmits fluid shear stress to the cortical cytoskeleton through the core proteins^7^. These shear stresses are believed to trigger specific cell signaling processes, such as cytoskeletal reorganisation and the production of nitric oxide^8^. Moreover, it is also known that docking of plasma-derived particles/molecules can lead to a number of changes in the local environment^6,9–15^. Thus, in order to understand these processes and functions, one needs a clear model of EG at the molecular scale.

Although EG has been studied for at least 50 years, a number of questions related to its molecular organisation remain unanswered. While the overall composition of the EG (as represented by its membrane and plasma proteoglycans, glycoproteins, and hyaluronic acids), is reasonably well known^16^, there are still several gaps in our understanding of its spatial structure and topology^17^. A number of theoretical and computational models have been developed to describe the mechanical and electrochemical properties of EG. One of the crucial steps in the construction of such descriptions was made by Squire and coworkers, who used an autocorrelation technique and Fourier transforms to analyse electron micrograph images of the EG. Their results suggested the hexagonal bush-like structural model of EG widely accepted today^18^. In their model, bushes of EG were linked through an underlying cortical cytoskeleton (CC), with a typical spacing of 100 nm between the “roots”. The luminal ends of EG chains (which can sense the flow of blood and interact with solutes), can then transmit torque to the CC through this connection. In addition, Weinbaum *et al*. have shown that with a bush-like structure, the leverage applied by the shear stress on the EG fibres may cause the deformation of the CC, which is believed to trigger signaling processes. In contrast, viscous drag on a single core protein is insufficient to produce such a deformation^19^. Another argument in favor of the bush-like structure of EG is that significant internal density variations are commonly observed in both the lateral and transverse directions, which is not consistent with the brush-like single filament picture^20–22^. Moreover, higher density clusters might play an important role in explaining the EG’s resistance to impacts from RBCs. Therefore, the general idea of a bush-like EG structure seems to be the most plausible, and is consistent with density autocorrelation measurements for tissues prepared using perfusion fixation and tannic acid treatment^23^. However, even in the most recent computational studies, the EG is still often modeled as a polymer brush comprising individual polymer chains or cylinders directly attached to the membrane. These works use brush relaxation after RBC impact and EG permeability data to parameterise the model. An all-atom model was proposed by Cruz-Chu *et al*.^24^, but despite the model’s high level of detail and high fidelity, their model is unsuitable for imitating large-scale and long-time properties of the EG due to immense computational costs. At the more coarse-grained level, the EG was also modeled as a part of the endothelium complex in a one-dimensional model^25^, and various methods have been employed in the numerical simulations of the atomistic and coarse-grained EG models, including Monte Carlo^26^, Molecular Dynamics (MD)^24^, and Dissipative Particle Dynamics methods^27^.

Up to now, the mechanics of bush-like structures have not been studied in detail, even in computer simulations. As a result, a measure of just how much the ultrastructure influences the elasticity and permeability of the EG was unknown. In this work, we implemented several variations of the bush model and attempted to quantify the differences between the bush and simple brush structures, and sought to determine the dependence of the mechanical properties of both models on the characteristics of the individual fibres. It is important to stress that in previous simulations, the elastic properties of individual fibres and the EG grafting were tuned to reproduce the EG height relaxation times using the simple brush presentation and elastic filament model^27–29^. However, in general, the parametrisation of the elasticity of individual EG fibres may be a non-transferable property. In addition, in many of the previous modeling works, thermal fluctuations were either neglected or artificially suppressed by imposing a low temperature in order to reduce the noise in the measurable quantities. Here, to assess the role of fluctuations, we tested the EG models with different ultrastructures and different elasticity-to-noise ratios.

In particular, to deduce the relation between the EG structure and its mechanics, we compared different models in terms of their density distribution and elastic response to indentation by a spherical bead, thus imitating atomic force microscopy (AFM) experiments. Several research groups have performed AFM tests on the EG and used various theories to interpret the results. However, the experimental values of the elastic constants, as observed by different labs, do not appear to be fully consistent with each other^30–33^, at least within the previously proposed theories. With this in mind, we note that in the following sections, we tested the consistency of our predictions following from different sets of computational and experimental data.

The main findings of our work can be summarised as follows: given the length of the filaments, density, and the ratio of their bending energy to thermal fluctuations, the variation in density and the surface roughness of the EG layer are expected to yield significant effects. In particular, parts of the layer with an average density of less than 50% of the grafting density may be over 150-nm-thick for a 400-nm-tall EG. Therefore, the EG brush should not be considered as a uniform density layer with a single elastic constant, and moreover, this conclusion refers to any EG model or polymer brush with long filaments. Secondly, we propose an elasticity model that takes thermal fluctuations and the non-uniform density distribution of the glycans into account. Our model predicts force profiles that are consistent with simulation results and experimental observations.

## Results

**Figure 1:**
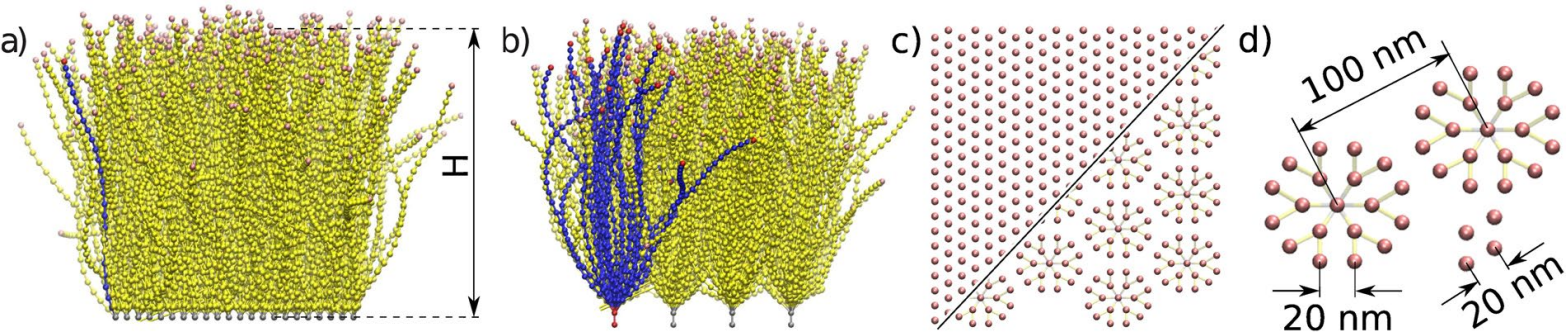
(**a**) Brush and (**b**) bush models of the EG. Single units are colored in blue, while the top and bottom (root) monomers are colored in red (tips and roots). (**c**) Initial configurations for the brush and bush models (combined picture), as seen from above. (**d**) Characteristic lengths between the adjacent beads, roots, and individual strains. The contour length of the filaments is 500 nm, while the equilibrium layer height *H* is about 400 nm.

In this work, we report on the mechanical properties of several models of the EG. Using coarse-grained molecular dynamics simulations, we investigate both flexible (FBr) and semi-flexible (SFBr) polymer brushes and bush-like structures (SFB) with three different values for the number of side chains in the bush (see Fig. 1). The EG fibres are represented by 500-nm-long bead-spring chains, where in the FBr case, each chain is tethered to the surface of the wall with just one fixed monomer, while in the SFBr case, the first two monomers are fixed in space so that the preferred direction of the subsequent bond is vertical (*i.e*., normal to the membrane). All fibres in the brush, and the central fibres in the the bush structures, are arranged hexagonally. Further details regarding the structure of the glycocalyx models are provided in the following Methods section.

### Monomer density distribution

**Figure 2:**
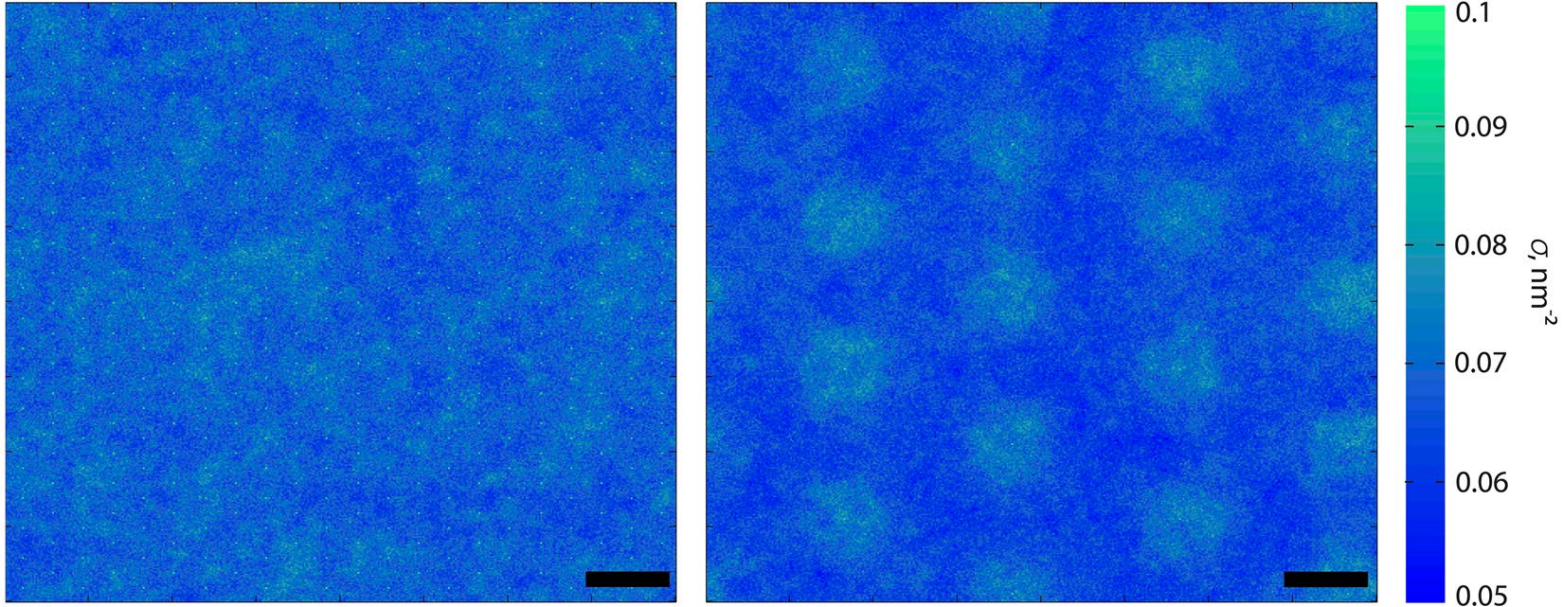
Monomer in-plane density distribution in brush (left) and bush-like (right) EG models. In the case of the bush-like model, the distribution is less uniform and there are local density minima and maxima. The scale bar is 50 nm.

As we stated in the Introduction, we expect several significant differences between the simple brush and bush-like structures. One such difference is the resulting permeability of the layer to plasma solutes, including protein globules. For this property, the distribution of EG beads across the *xy*-plane is of extreme importance and a greater uniformity of this distribution would presumably mean a stronger depletion of smaller particles from the layer. At the same time, the presence of regular higher density clusters could help the EG resist the impact of RBCs. Thus, in order to quantify the density inhomogeneities associated with the bush structure, we measured the monomer density distribution across the layer (Fig. 2). As one can see from the figure, the distribution in the case of bush-like structures does indeed show areas of lower and higher densities. Therefore, even if the distributions along the *z*-axis appear to be identical, the permeabilities of different structures may be affected.

**Figure 3:**
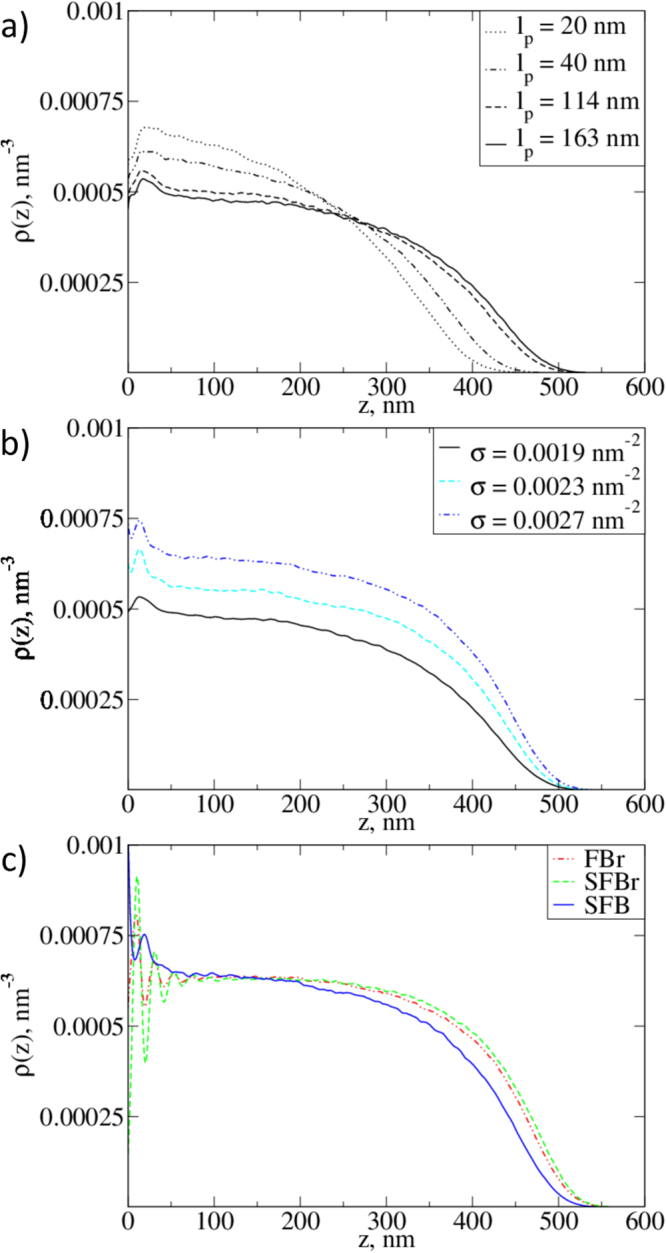
Monomer density distribution in bush- and brush-like EG structures as a function of distance from the membrane, as obtained from simulations. (**a**) Monomer density distribution in the EG for different fibre persistence lengths. (**b**) Density distributions in the EG for different grafting densities. (**c**) Density distributions for different structures with the same fibre persistence length and grafting density. The parameters of the semi-flexible bush model were *σ* = 0.0019 nm^−2^ and *l*_*p*_ = 163 nm.

We also collected statistics on the glycan bead positions along the normal to the membrane. The curves in Fig. 3 show the glycan density distributions inside the EG. These distributions are expressed in terms of the monomer number density along the *z*-axis as a function of distance from the impenetrable membrane at *z* = 0. In agreement with previous observations, all the curves obtained for different fibre persistence lengths, grafting densities, and EG structures have the same characteristic shape, consisting of a parabolic part and a sigmoidal tail^34–38^. The equilibrium height of the layer in our model varies from 400 nm to over 500 nm, while the fibre contour lengths, *L* = 500 nm, are the same for all curves. As expected, the height of the brush increases with increasing persistence length *l*_*p*_, as the fibres acquire a straighter conformation. For *l*_*p*_ = 20 nm and 40 nm, we see that the curve is predominantly parabolic in shape (Fig. 3a), whereas for relatively rigid chains, the distribution is closer to a smooth step function. We also note that the difference between the *l*_*p*_ = 114 nm and *l*_*p*_ = 163 nm curves is significantly less than that between the *l*_*p*_ = 20 nm and *l*_*p*_ = 40 nm curves, and between the *l*_*p*_ = 40 nm and 114 nm curves, which implies that the shape is less sensitive to the fibre persistence length for rigid chains, where *l*_*p*_ is comparable to the contour length.

The grafting density of the chains, *σ*, is the primary factor determining the glycan density distribution, *ρ*(*z*) in the EG (Fig. 3b). As outlined above, we modeled the SFB systems using three different side chain numbers, which each correspond to different grafting densities. From the data presented in Fig. 3b, we see that the shape of the distribution of monomers inside the EG does not change significantly with the variation of the number of chains per unit area. Moreover, we observe a proportional increase in *ρ*(*z*) and a corresponding increase in the maximum height of the layer with increasing *σ*.

In order to assess the effect of the EG’s ultrastructure, we compared the vertical density profiles, *ρ*(*z*) of the three SBr, SFBr, and SFB models (Fig. 3c). Since the grafting density was approximately equal for all three cases (*i.e*., a difference of less than 5%), the changes in profiles therefore reflect the qualitative differences between these models. We can see from the figure that the density in both the SBr and SFBr systems seems to be more uniform in the bulk of the layer than in the SFB model, which leads to a steeper drop in density at the top of the EG. Furthermore, it is clear that the SFBr model exhibits the sharpest density drop. The difference between the density profiles in the bottom (small *z*) region is caused by the difference in the structural organisation of the EG close to membrane (see Fig. 1a and b).

Shifting the functions for different fibre persistence lengths in order to match their minimal values at the same position also allowed us to compare the shape of the distributions in the upper (luminal) region (see Fig. S3 of the Supplementary Information). We found that the upper parts of the profiles are essentially insensitive to the bending rigidity of the fibres, *i.e*., they have the same values and slopes for EGs of the same grafting density. At the same time, variation of the grafting density shows a clear influence on the shape of the profile *ρ*(*z*) in the upper region.

Then to provide a more quantitative description of the density profiles, we fitted the data with piecewise-smooth polynomial functions. The density distribution histograms naturally split into two convex and concave parts, separated by an inflection point. This division appears to reflect the change in the prevailing forces inside the layer at different depths: the deeper part seems to be dominated by the usual parabolic term that is associated with the excluded volume, while the upper part results from an interplay of thermal motion and fibre elasticity. We found a single polynomial form that accurately describes both top and bottom regions of the density profile *ρ*(*z*) using a combination of parabolic and soft-step functions

1 $$f(z)={c}_{1}{z}^{6}+{c}_{2}{z}^{5}+{c}_{3}{z}^{2}+{c}_{4}$$

The density profiles could then be fitted using this function with two sets of coefficients *c*_1_ − *c*_4_ so that the values *f*(*z*) matched at the inflection point. These values are collected in Table 1 of the Supplementary Information. Studying the coefficients for different models and values of the bending rigidity, we found that the values of the coefficients are influenced by both the grafting density and the fibre persistence length. It is intuitively clear that the stiffness of the top layers is less than that of the bottom layers, and consequently, the coefficients are smaller in the upper part of the curve. Later, we use this function to find a closed-form expression for the force exerted on the AFM tip via the indentation depth.

### Elastic response

**Figure 4:**
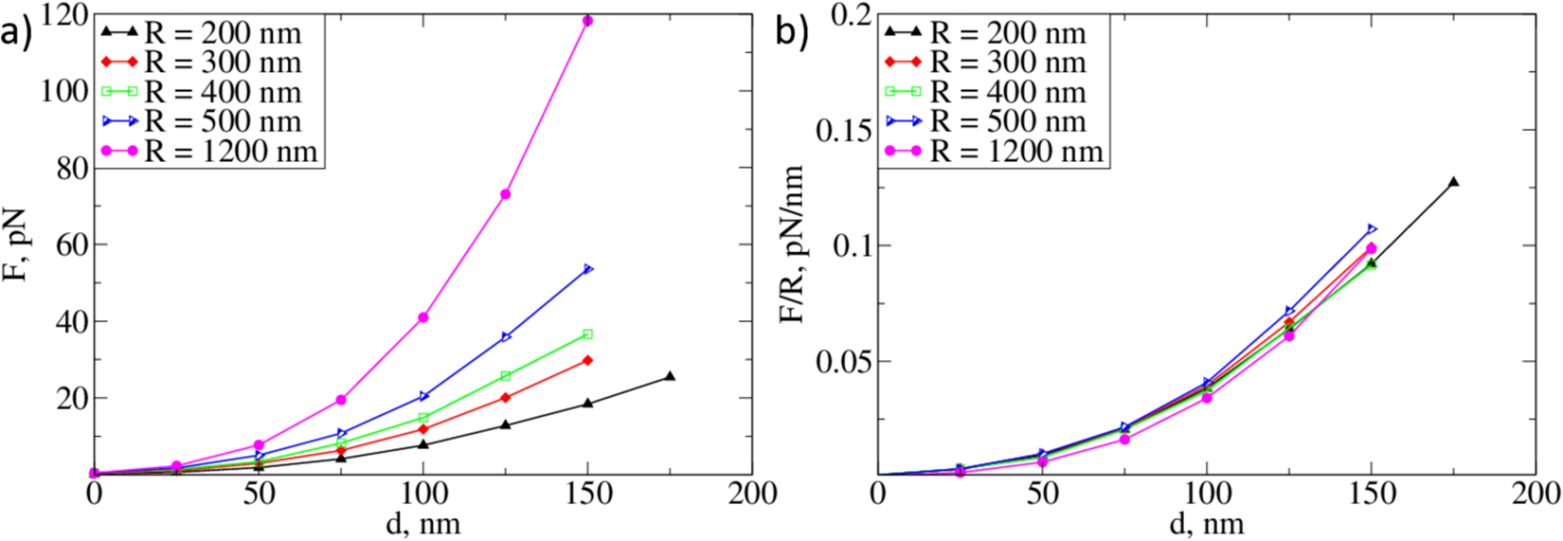
(**a**) Reaction force *F*(*d*) for the SFB EG model with 19 chains per bush, *σ* = 0.0019 nm^−2^, and *l*_*p*_ = 163 nm for different AFM tip radii, as obtained from our simulations. (**b**) The scaled reaction force demonstrates the linear dependence of the force on the radius of the AFM tip for small indentations.

To assess the elasticity of the different EG models, we imitated the indentation of an AFM tip into the layer by measuring the average vertical force on the tip as a function of its position with respect to the top of the EG for various different parameters of the EG (i.e., persistence length, grafting density, and structural organisation). We also sought to find the combination of these values that best fit the experimental data. The forces exerted on the AFM tips with radii *R* = 200,300,400,500, and 1200 nm were measured using the SFB model with 19 chains per bush (see Fig. 4a). The predicted forces ranged from 8 pN to 42 pN at an indentation depth of 100 nm for different tip radii. All the curves exhibit the same non-linear shape as a function of *d*, and the force increases proportionally to the tip radius at small indentations. As one can see in Fig. 4b, the values of the force scaled by the tip radius *F*(*d*)/*R* lay on top of each other for all radii in the 0–100 nm range.

We note, however, that our simple models may have underestimated the EG elasticity, as we neglected some essential structural elements such as hyaluronans, which are known to cross-link the glycan fibres. Thus, in order to estimate their contribution to the elasticity, we introduced additional chains mimicking hyaluronic acids into our model. These additional chains were placed inside the EG horizontally, then relaxed and connected with harmonic bonds to the nearest beads of the EG fibres. The data extracted from these simulations with transverse chains revealed stronger repulsive forces acting on the tip at the same indentation depth. The dependence of the measured force on the tip radius, however, remained linear (see Figs S5 and S6 of the Supplementary Information), and this dependence was observed for both brush, FBr and SFBr models, and the bush SFB model. We note that this observation does not agree with previous statements based on the Hertz theory^30^, which predicts that the force scales as *F* ∝ *R* ^3/2^ (below we show that this linear dependence should hold up to the micrometer scale). However, as the Hertz theory considers continuous materials with uniform density, this discrepancy might indicate that the Hertz theory approximation does not hold for EG, at least at low grafting and cross-linking densities. In addition, a further contribution to the overall elasticity of EG may come from the solutes, which can yield a significant contribution to the effective volume fraction, however, we did not study this effect here.

**Figure 5:**
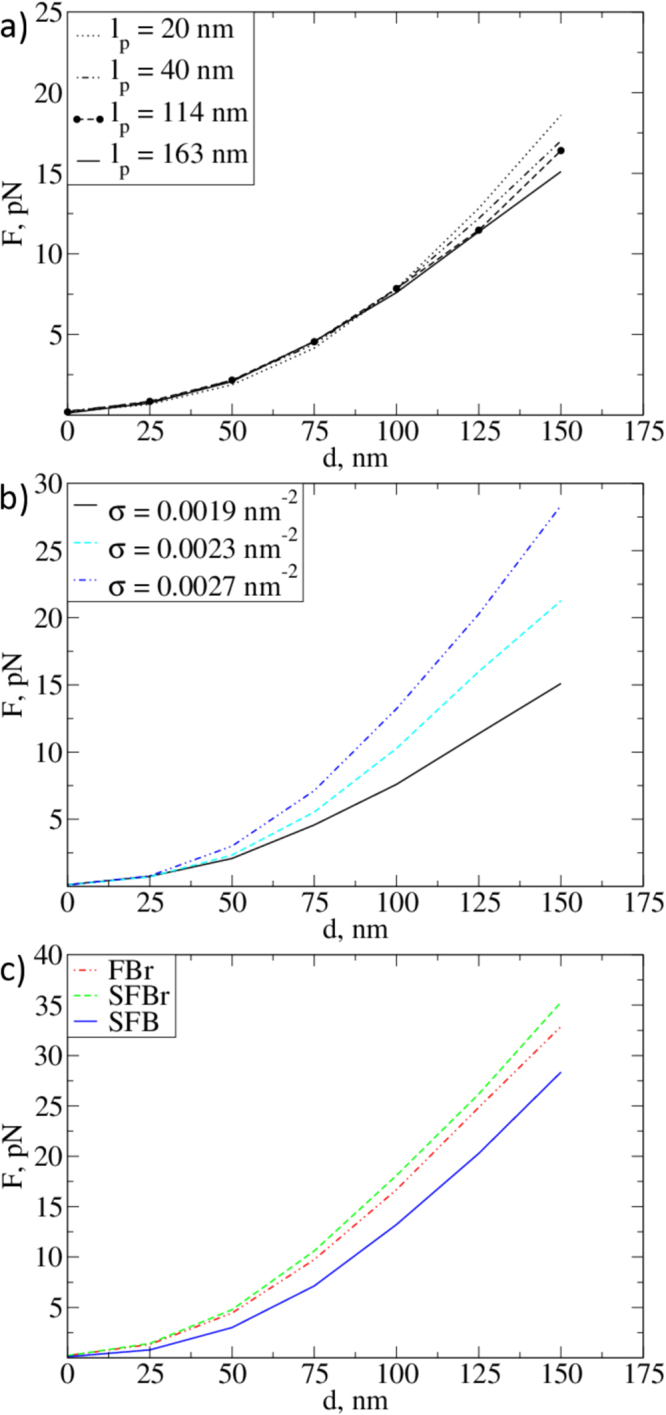
Force-indentation curves for the deformation of different calyces. (**a**) Reaction force of an SFB EG for *σ* = 0.0019 nm^−2^ with different fibre persistence lengths; (**b**) Reaction force of an SFB EG with *l*_*p*_ = 163 nm at different grafting densities; (**c**) Forces for different EG models of the same grafting density *σ* = 0.0019 nm^−2^ and *l*_*p*_ = 163 nm. Radius of the indenter is taken to be 200 nm.

In order to study the impact of the persistence length, grafting density, and structure of the model on the reaction force exerted on the indenter, we performed a series of simulations with a spherical tip with radius *R* = 200 nm. We found that the reaction force is insensitive to the bending rigidity of the fibres at small indentations, and in similar fashion to the observed density distributions, all the measured force curves have the same values in the range 0 to 100 nm (Fig. 5), and only start to diverge for *d* > 100 nm. Interestingly, for the larger persistence lengths, we registered a slower increase of the force with the indentation depth at *d* ≈ 100 nm than for the less rigid brushes. This is related to the fact that a brush with more rigid fibres is more stretched along the vertical axis, and the monomer density increases more slowly with the indentation depth than for a brush with softer fibres (see Fig. 3a). As expected, increasing the grafting density leads to a proportional increase of the reaction force (Fig. 5b). This dependence suggests an ideal gas-like pressure law, which predicts a linear relationship between the force exerted on AFM tip by the EG and its density distribution. Moreover, for brushes consisting of individual chains (FBr, SFBr), we observe larger forces than those for the bush-like structures (SFB) at the same grafting density. This result may be understood provided that there is some flexibility of the side chains and in the regions of lower flexibility in the bush-like EG structures. Individual fibres in the bush-like structures can then fall to the bottom of the EG layer (note the density peaks in Fig. 3) and make the lower layers stiffer while making the upper ones less dense and more penetrable.

Using the experimental data from ref.^30^ we found the force on an AFM tip of radius *R* = 1.2 *μ*m at an indentation of 100 nm from the point of initial contact to be about 42 pN. The *in vitro* experiment by Marsh and Waugh was designed to measure the thickness and Young’s Modulus of the glycocalyx. A flow chamber was constructed so that human umbilical vein endothelial cells (HUVECs) could be grown under a shear of 1.0 Pa (10 dyn/cm^2^) at 37 °C and subsequently transferred to the atomic force microscope. A tipless AFM cantilever then picked up streptavidin beads (with a diameter of 2.4 *μ*m) and was used to indent the samples after being suitably calibrated. Twenty indentations were repeated at a rate of 1 *μ*m/s, with an interval of 6 seconds being taken between the repetitions. In our simulations, we achieved the closest agreement with the AFM data for a SFB EG model with 19 chains per bush, where the exhibited reaction force was approximately 41 pN at an indentation of 100 nm and with an AFM tip of radius 1200 nm. Assuming that the dependence of the reaction force on the radius of the AFM indenter is linear, we can predict that the reaction force for a 200-nm sphere is six times less than that of a 1200-nm one. Our simulation results are in good agreement with that assertion, as we found the reaction force for the smaller tip to be 7 pN. For all values of the persistence length we considered, our model yielded a force of 7 pN to 8 pN at an indentation depth *d* = 100 nm. The volume fraction of this bush-like EG is *c*. 8.6%, which is almost half that proposed by Weinbaum^19^. We note that we also varied the stiffness (bending rigidity) of the harmonic spring bonds *J* to improve the fit, but for realistic inter-particle potentials we could not bring the reaction force *F* below 6–8 pN. We conclude that the excluded volume interactions of monomers is the main factor responsible for the compressibility of the EG. Finally, we note that while an alternative way to match the experimental force-indentation curves would be to lower the grafting density, this method would require a significant departure from the hexagonal structure observed by Squire and from the model proposed by Weinbaum^19^.

### Model of the force-indentation relationship

The similarity between the observed density distributions and the reaction force-indentation curves for different EG structures suggests the existence of a universal elasticity model for all structures. In order to relate the density distribution to the force, we assume that the force on the indenter originates from contact with glycan monomers. Due to the axial symmetry of the system, one can expect that the average reaction force has only a vertical component. As the upper part of the EG is quite dilute (the density is typically below 4%), we can estimate the net force using the ideal gas approximation. We assume that the normal pressure on any surface element of the AFM tip is proportional to the mean number of contacts of the glycan beads with the tip at a given height, and then calculate the vertical projection of the resulting force. We therefore propose that the reaction force is only due to the thermal motion of glycans and is not significantly affected by the fibre’s elasticity (i.e., interactions along the fibre or between the fibres). We also neglect the possible deformation of the membrane, which can be verified *a posteriori*. Obviously, the pressure on the AFM tip varies with the indentation depth and the density distribution of the affected part of the EG. With these approximations, we find that the vertical reaction force *F* may be written as:

2 $$\begin{array}{c}\varphi (d)=\arcsin \frac{\sqrt{{R}^{2}-{(R-d)}^{2}}}{R}\\ z=H-d+R(1-\,\cos \,\theta )\\ P(z)=k\rho (z)\\ F=2\pi {R}^{2}{\int }_{0}^{\varphi }P(z)\,\cos \,\theta \cdot \,\sin \,\theta \,d\theta \end{array}\}\begin{array}{c}F(d)\,\approx \,\pi k\rho (H)d(2R-d)-\frac{\pi k{d}^{2}}{6}f(d),\end{array}$$

where

3 $$\begin{array}{ccc}f(d) & = & {c}_{3}(d)(12HR-4d(H+R)+{d}^{2})\\  &  & +\,2{c}_{2}(d)(15{H}^{4}R-5d{H}^{3}(H+4R)\\  &  & +\,5{d}^{2}{H}^{2}(H+3R)-3{d}^{3}H(H+2R)+{d}^{4}(H+R))\\  &  & +\,3{c}_{1}(d)(12{H}^{5}R-4d{H}^{4}(H+5R)+5{d}^{2}{H}^{3}(H+4R)\\  &  & -\,4{d}^{3}{H}^{2}(H+3R)+2{d}^{4}H(H+2R)).\end{array}$$

**Figure 6:**
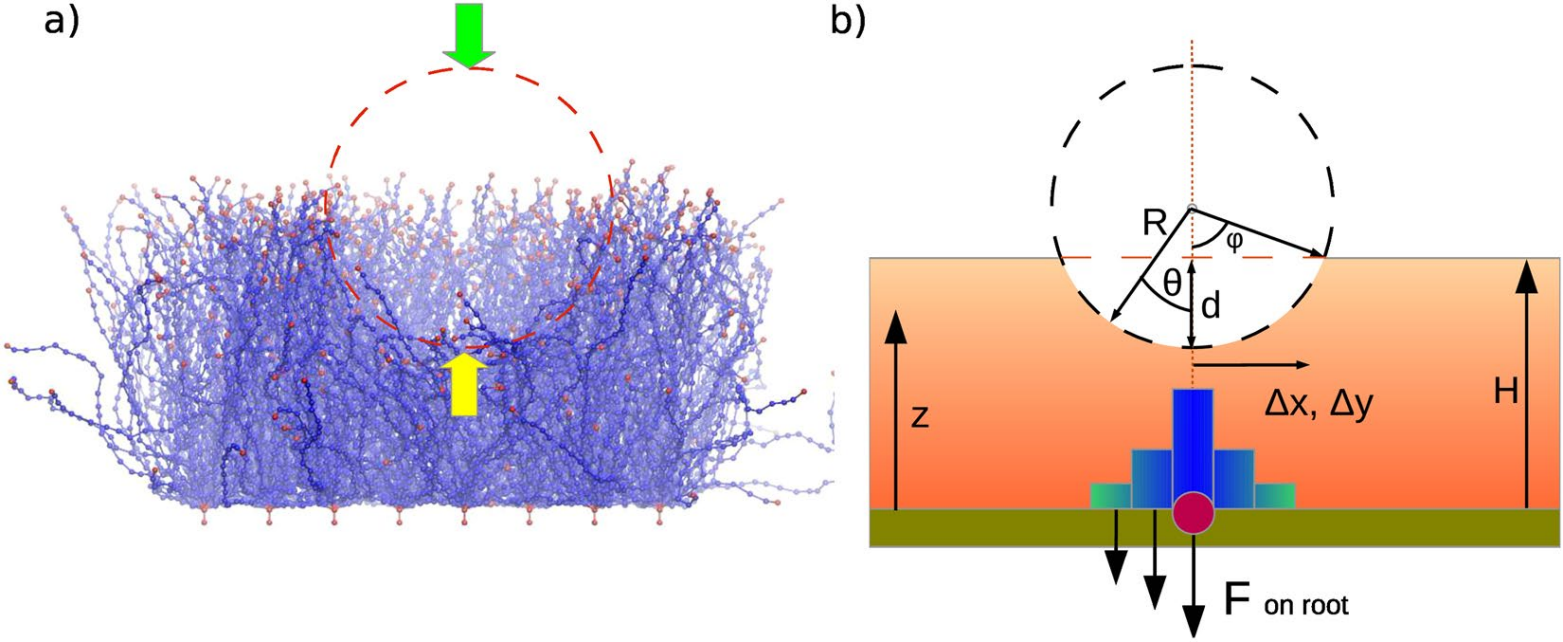
(**a**) Snapshot from a simulation of the AFM experiment. The green arrow indicates the direction of the force from the EG acting on the tip of the cantilever. The yellow arrow indicates the reaction force exerted on the tip by the EG. (**b**) Graphical scheme representing the applied forces and how they were calculated.

**Figure 7:**
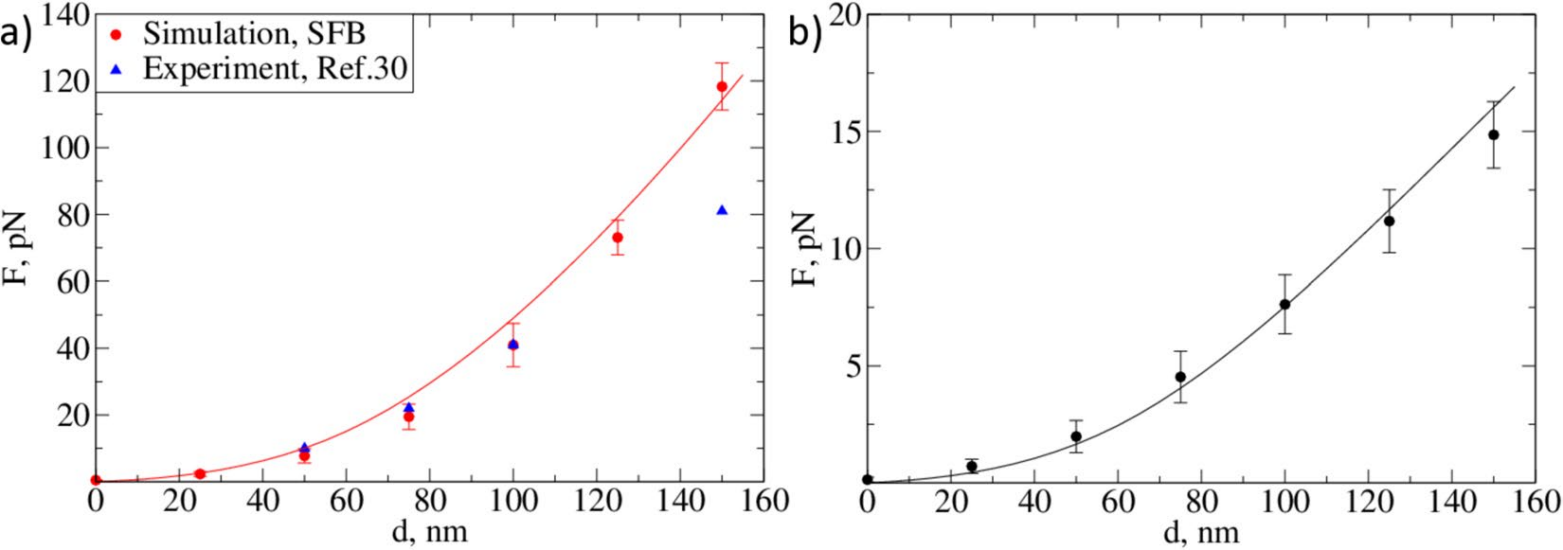
(**a**) Simulated and experimental EG reaction force for a tip with radius *R* = 1.2 *μ* ^30^. Simulation results were obtained using an SFB model with *l*_*p*_ = 163 nm and *σ* = 0.0019 nm^−2^. The solid line indicates the curve obtained from Eq. (2) with *k* = 0.67 pN · nm. (**b**) Simulated EG reaction force for a tip of radius *R* = 200 nm. The solid line is the predicted dependence obtained from Eq. (2) with *k* = 0.67 pN · nm. Further examples are included in the Supplementary Information.

In Eq. (2), *ϕ* is the maximum angle formed by the bottom point of the tip, its centre, and the point on the surface of the sphere where it touches the surface of the EG, while *θ* is the polar angle and varies between 0 and *ϕ*. The monomer density at the top of the EG, *ρ*(*H*) = *ρ*(*z* = *H*), is given by Eq. (1), where the definition of *H* is discussed below. Terms of higher order in *d*/*R* and *d*/*H* are omitted in Eq. (3). For clarity, all the variables are depicted in Fig. 6b. At small indentations, $d\ll R$, the leading term of the force is *F*(*d*) ≈ 2*πkρ*(*H*)*dR*. However, as was noted earlier, the coefficients *c*_*i*_ take different values in the two different density fitting regions. By adjusting the coefficient *k*, one can tune *F*(*d*) to best fit data from experiment and simulation Fig. 7.

Thus, to predict the magnitude of the force for a particular system with a known monomer density distribution, one needs to consider the value of *ρ*(*z*) between the point defined as zero indentation, *d* = 0, and the point of desired maximum indentation. The profile can then be combined with the function *F*(*d*), Eq. (1), and the final expression relates the force *F*(*d*) to the radius of the tip *R*, and the coefficient *k*, which represents the stiffness of the EG layer. As per our earlier assertion, the leading force term depends linearly on the tip radius *R*.

**Table 1:** Model parameter nomenclature and abbreviations.

*B*	Second virial coefficient
*c*_1_, *c*_2_, *c*_3_, *c*_4_	Linear coefficients for *f*(*z*)
*d*	Indentation depth
*F*	Reaction force
*f*(*z*)	Density profile fitting curve
FBr	Flexible Brush model
*H*	Height of the brush, reference height for the indentation
*J*	Spring constant for harmonic bonds
*K*	Bending rigidity (torsional stiffness) of a filament
*k*	Elastic coefficient representing the stiffness of the brush
*k*_*B*_	Boltzmann constant
*L*	Contour length of the filaments
*l*_*p*_	Persistence length of the EG fibres
*N*	Number of chains in a single bush
*P*	Pressure exerted on the tip by the EG monomers
*ϕ*(*d*)	Maximum angle formed by the vertical to the bottom of the tip, and the point of the sphere where it touches the surface of the EG
*R*	Radius of the AFM tip
*r*_0_	Optimal length of the EG fibre segment
*ρ*(*z*)	Vertical monomer density distribution function
SFB	Semi-Flexible Bush-like model
SFBr	Semi-Flexible Brush model
*σ*	Grafting density, number of chains per cross-section unit area
*T*	Absolute temperature of the simulated system
*θ*	Polar angle for the point on the surface of the indented part of the sphere
*V*_*a*_	Bond-angle potential for EG fibres
*V*_*h*_	Harmonic spring potential for EG fibres
*V*_*LJ*_	Lennard–Jones potential
*W*	Work of compression of the EG

In order to fit the model to our simulated systems, we first identify the relevant range of vertical tip positions on the force curves and measure the average forces exerted on the AFM tip at different positions with respect to the membrane (bottom of the brush). We then start from a position well above the EG (*ca*. 50 nm above the layer) and keep lowering the tip until a non-zero average reaction force (*F* > 0.1 pN) is detected. From this point on, we see that the force continuously increases as the tip penetrates into the brush, and we take this initial point of contact to be the zero indentation position *d* = 0. Then, we map this point onto the monomer density distribution so that the bottom of the tip is at *z* = *H*. Thus, all the monomers residing above this point are ignored, as they do not contribute to the average repulsion force. We then fit the force curves up to an indentation of 150 nm from our simulation or experimental results to determine the coefficients (see the example in Fig. 7 and Table 1 of the Supplementary Information). For the simulated SFB model with the lowest density, *σ* = 0.0019 nm^−2^, we find that *k* lies in the range 0.67–0.68 pN · nm. For higher densities, the coefficient increases to *k* = 0.8 pN · nm and 0.87 pN · nm, for *σ* = 0.0023 nm^−2^ and 0.0027 nm^−2^, respectively. The results for the FBr and SFBr brushes yield somewhat greater elastic coefficients: *k* = 0.9 pN · nm and *k* = 0.96 pN·nm at *σ* = 0.0027 nm^−2^, respectively. Fitting the experimental data of ref.^30^ up to *d* = 150 nm (see Fig. 7a) yields a lower value of *k* = 0.47 ± 0.03 pN · nm. The deviation of the force from the trend observed at smaller *d* indicates that at the deeper indentations the AFM tip starts to probe other elastic response mechanisms, such as deformation of the cell membrane.

**Figure 8:**
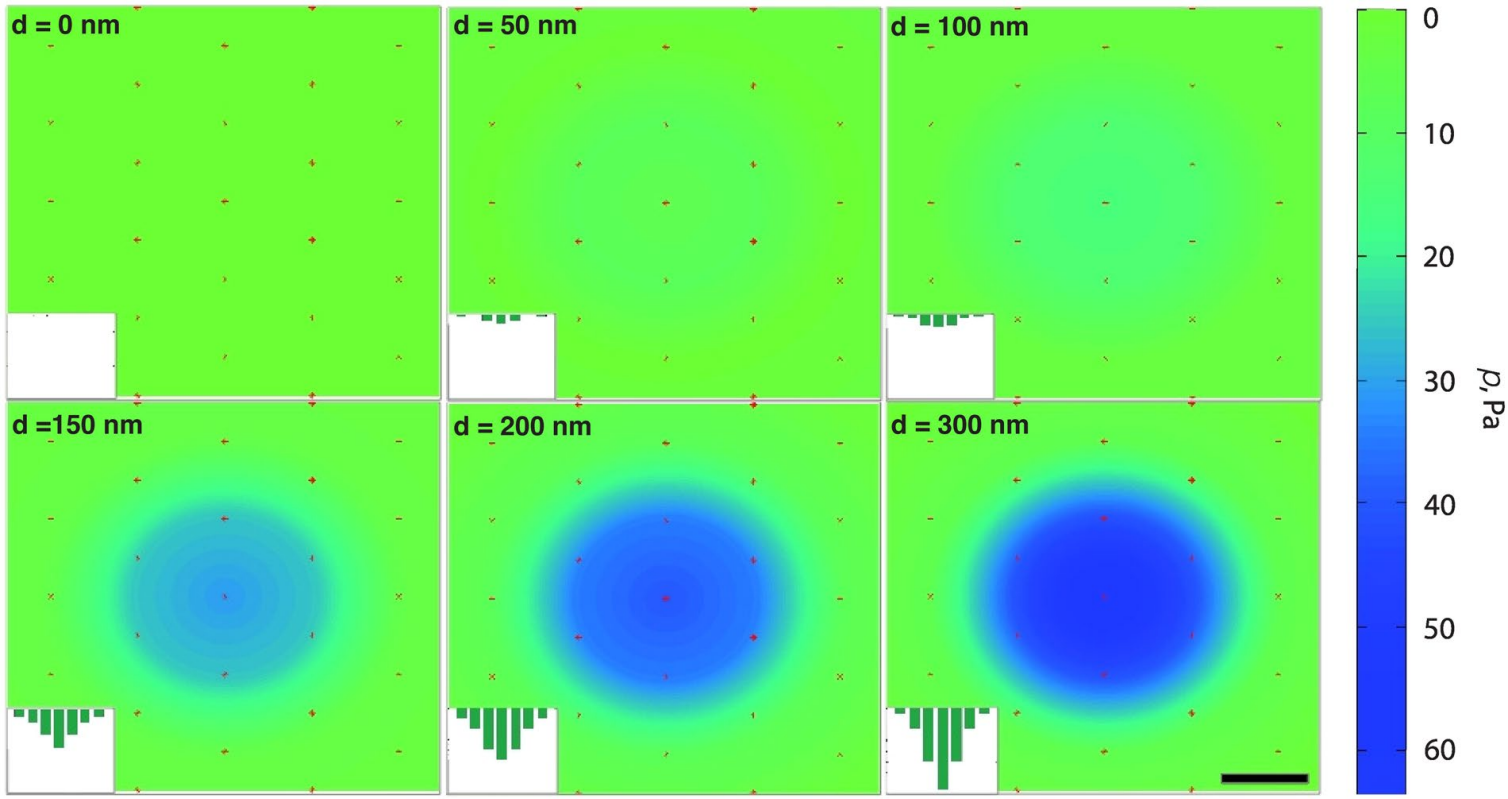
Pressure on the cell membrane for different indentations with a 400-nm AFM tip. Semi-flexible bush model parameters were as follows: *σ* = 0.0019 nm^−2^, *l*_*p*_ = 163 nm. The scale bar is 100 nm.

To check this hypothesis, we sought to determine the maximum indentation, at which we can neglect the deformation of the membrane, since at large indentation depths, the force will be transmitted to the membrane and will deform it^32^. At such a point, the membrane will yield and the *F*(*d*) dependence will become weaker. We measured the force transmitted to the roots of the bushes in a simulated AFM experiment and found that at indentations of less than 100 nm, the pressure on the membrane does not exceed 15 Pa. Moreover, the overall force (0.4 pN) appears to be smaller than the sensitivity of the AFM^39^ at indentations of up to 100 nm, as shown in Fig. 8. As the membrane deformation is not included in the model (the support is rigid), the force values measured in simulation at *d* > 100 nm follow the same trend as for smaller indentations, so we find the same high elastic constant for all values of *d*.

Apart from the EG density distribution and the forces exerted on the tip, the forces applied to the bottom constraint and to the bush roots were measured, and our methodology led us to identify a relationship between the structure of the EG, its density distribution, and the reaction force exerted on the AFM tip by the EG. Previous AFM experimental studies of the EG found a qualitative change in the reaction force at indentations of greater than 100–150 nm^30,32^, and it was suggested that a deformation of the underlying cell membrane occurs at these indentations that contributes to the overall reaction force. We measured the forces transmitted to the root particles of the EG and in our simulations, a qualitative change also appears at indentations of about 100–150 nm, which is in good agreement with the experimental data and shows that the range considered in our AFM simulations was chosen correctly.

## Discussion

Coarse-grained simulations of bush and brush models of the EG, as reported in this work, reveal qualitative similarities between these ultrastructures. Most importantly, both models demonstrate non-uniform monomer density distributions, such that the reduced density region (*i.e*., less than half of the mean density) spans over 20% of its maximum height. This property is related to an inherent feature of both models—the long fibres. Thus, for realistic values of the grafting densities and volume fractions, and with bending rigidities as proposed in the literature, these fibres must be quite flexible at distances of several hundred nanometers and their conformations must be strongly affected by thermal fluctuations. An EG brush made of 500-nm-long fibres would necessarily have a diluted luminal layer with a thickness of up to a hundred nanometers, independently of the ultrastructure. We stress that any model probing the mechanics of the EG must take this consideration into account. In particular, modeling the EG as a uniform elastic medium over the whole thickness cannot be justified.

We also found that the density distributions can be fitted by simple polynomial functions, and include a parabolic contribution which is observed in the dense parts of the EG (a common feature of most polymer brushes)^34–37^, and a sigmoidal part, which is detected in the upper luminal layers. This density distribution seems to be the most important factor affecting the EG elastic response. We see that the reaction force of a compressed or indented EG is not very sensitive to the fibre bending rigidity, but varies somewhat with the grafting density. Specifically, we found only a 10% increase of the force upon an 8-fold variation of the bending rigidity. Qualitatively, the ideal pressure model that we used to describe the reaction force seems to agree with both simulation and experimental force-indentation curves for small deformations. The elasticity coefficient in our model has the dimensionality of energy and lies in the range of 0.1 to 1.0 pN · nm. This number should be compared to the characteristic thermal energy *k*_*B*_ *T* ≈ 4.2 pN · nm at room or body temperature, and would be expected to be close to 1 *k*_*B*_ *T* for an ideal gas of monomers. However, the number of translational degrees of freedom in the EG is significantly less than the number of monomers. The number of free segments per fibre (*i.e*., the number of segments that can move independently), can be estimated as the contour length over the Kuhn length, *L*/(2*l*_*p*_). For the values of the persistence lengths we have studied here (20 nm to 163 nm), we would expect to have from 2 to 12 independent segments per chain. Therefore, the expected pressure coefficient must be in the range 0.1 *k*_*B*_ *T* to 0.5 *k*_*B*_ *T*, or equivalently, in the range 0.4 pN · nm to 2.0 pN · nm, which is indeed what we observe. We should note that the validity of the ideal pressure model is partly justified by the low monomer density in the luminal layer (typically less than 5% of the volume fraction). Further corrections to the model can be easily introduced for larger indentations, which should take monomer–monomer repulsion effects into account. In that case, the pressure can be evaluated as $P(z)=k\rho (z)+\frac{1}{2}B{\rho }^{2}(z)$, where *B* is the second virial coefficient.

The fits to AFM experiments are also in the indicated range. With very rigid fibres, one would ultimately expect to have just one degree of freedom per chain. At this point, however, the model would fail as a whole and the experiment would be sampling the rigidity of the fibres. In any case, the EG relaxation experiments do not agree with such a model, as they give a value of *EI* = 490 pN · nm^2^ ^17^, which corresponds to a persistence length of about 114 nm for individual fibres, notably less than their contour length. Moreover, one cannot expect chains of this length and rigidity to keep a normal orientation with respect to the cell membrane along the whole length. In fact, the bush-like model (SFB) comprising the core fibre and 19 side chains (and thus having an average monomer volume fraction of about 8%), showed the best agreement with experimental data within our proposed model. We should note that any model with a higher volume fraction would be too rigid and inconsistent with the AFM measurements.

We also found that with both types of ultrastructure, only small normal forces are transmitted through the EG to the root particles, *i.e*., to the membrane. Consequently, a significant deformation of the endothelial cells after penetration of the AFM tip can only be expected at depths of about 100 nm (or greater) from the membrane.

Our simulations show that the reaction force from the weakly-deformed EG is proportional to the tip radius, and this dependence can be used to test the theory and predict the force from indenters of different sizes. We found that the linear dependency of the model remained even when transverse chains in the EG were included to imitate hyaluronic acids (see Figs S5 and S6 of the Supplementary Information). We should also emphasise the fact that the density and elasticity of the EG make it difficult for foreign particles to penetrate the layer by brute force. In particular, the work required to indent the EG by 300 nm, $W={\int }_{0}^{d}F(s)\,ds$, exceeds 1000 *k*_*B*_ *T* for *R* = 500 nm particles, and exceeds 300 *k*_*B*_ *T* for *R* = 200 nm particles (see Fig. 6). Therefore, drug delivery systems should use a smarter strategy if endothelial cells are to be targeted. In contrast, the required energy for smaller particles would be much lower as the required work scales nonlinearly with their size. One could therefore expect a figure of a few tens of *k*_*B*_ *T* for a nanoparticle of radius 20 nm. This result suggests that a possible penetration mechanism could be enabled via attractive interactions between the nanocarrier and the glycans, such as electrostatic attraction for positively charged carriers.

Finally, we note that the difference between the brush and bush-like models is not qualitative but quantitative, and appears to be significant for fitting experimental AFM data. Moreover, the fixed perpendicular orientation of the fibres seems to add extra rigidity to the layer. However, we could not reproduce the elastic response of real EG layers with the SFBr model with any proposed combination of bending rigidity, volume fraction, and grating density from the literature. Another argument in favour of the bush model is the difference in torques resulting from the drag forces applied to one chain (or to the bush) in blood flow. This difference may have a significant impact on the cell signaling processes. We suggest that the permeability of EG to foreign nanoparticles may also be affected by the greater irregularity represented in the bush-like coating. This part of the work will be reported in a subsequent article.

## Methods

We used a coarse-grained model of the EG comprising homopolymeric bead-spring chains grafted onto a uniform substrate. We neglected any deformations of the cell membrane and modeled it as a solid impenetrable surface. The EG was modeled in several distinct realisations: (I) individual semi-flexible filaments directly attached to the membrane and with perpendicular orientation (SFBr); (II) individual semi-flexible filaments attached to the membrane without preset orientations (FBr); and (III) bush-like structures where only the core filament was attached to the surface, while the branches were attached to the core (SFB). All the core filaments and branches were considered to have the same bead-spring chain structure and are as illustrated in Fig. 1.

In all three models, we considered the glycan fibres to be made up of 25 monomers each (giving a contour length of *L* = 500 nm), thus yielding an equilibrium brush height in the range 400–500 nm, which corresponds to the most common experimental system of capillary EG in rats^40^. In the case of the SFB model, we used *N* = 19, 23, or 27 polymer chains per bush, which corresponded to grafting densities *σ* of 0.0019, 0.0023 and 0.0027 chains per nm^2^, respectively. Each bush consisted of the core chain and *N* − 1 linear polymer chains that branched from it at a distance of 20 nm from the bottom. These side chains were able to rotate freely around the point of attachment to the core. Both core and side chains consisted of beads of radius 6 nm, representing saccharide groups, and a spacing of 20 nm was used between adjacent beads^18,19^.

In order to calculate the density distributions of the EG beads, we ran molecular dynamics (MD) simulations for 225,000 cycles (with a timestep of 0.005 simulation units). Before collecting the data, the EG configurations were equilibrated for 100,000 MD steps, where the relaxation of the position of the centre mass of the top beads was used as the criterion for equilibration. As a standard reference, we chose the 19-chain bush model and performed four series of simulations with bending rigidities of 85, 170, 490, and 700 pN · nm^2^. These rigidities corresponded to persistence lengths of 20, 40, 114, and 163 nm, respectively. For this type of EG, the initial volume fraction (*i.e*., taken before the equilibration stage) was 8.5%. However, we note that we also considered bushes with 23 and 27 chains, which corresponded to volume fractions of 13% and 16%, respectively. The considered brush structures FBr and SFBr also had an initial monomer volume fraction of 16%. Aside from the reference SFB model, all models were only considered for the cases with persistence lengths of *l*_*p*_ = 114 nm and *l*_*p*_ = 163 nm.

In the construction of our simulation box, we used periodic boundary conditions in two lateral dimensions (*x* and *y*) and impenetrable constraints for both liquid and the glycan beads at the membrane, with the normal lying along the *z*-axis. The EG filaments were modeled by bead-spring chains with the beads representing the monomers and the springs representing a repulsive Lennard–Jones (LJ) potential connected by harmonic bonds. Interactions between the monomers were determined by *σ*_LJ_–the sum of their radii, *ε*–the strength of interaction, and *r*–the distance between the centres of the beads. The potential was given by

4 $${V}_{LJ}(r)=\{\begin{array}{cc}4\varepsilon ({(\frac{{\sigma }_{{\rm{L}}{\rm{J}}}}{r-{r}_{{\rm{o}}{\rm{f}}{\rm{f}}}})}^{12}-{(\frac{{\sigma }_{{\rm{L}}{\rm{J}}}}{r-{r}_{{\rm{o}}{\rm{f}}{\rm{f}}}})}^{6}+{c}_{{\rm{s}}{\rm{h}}{\rm{i}}{\rm{f}}{\rm{t}}}), & {r}_{min}+{r}_{{\rm{o}}{\rm{f}}{\rm{f}}} < r < {r}_{{\rm{c}}{\rm{u}}{\rm{t}}}+{r}_{{\rm{o}}{\rm{f}}{\rm{f}}}\\ 0, & {\rm{o}}{\rm{t}}{\rm{h}}{\rm{e}}{\rm{r}}{\rm{w}}{\rm{i}}{\rm{s}}{\rm{e}}\end{array}$$

Here, *r*_off_ is the offset distance for the LJ potential and *c*_shift_ is the energy of the minimum configuration. The interaction potential was cut at the minimum and then shifted so that the cut-off point corresponded to the zero value. Full model parameter nomenclature and definitions are presented in Table 1.

In order to consider long times in our simulations, we replaced the LJ potential between the glycan beads by a much softer “hat” potential. This replacement allowed us to employ a larger time step while preserving the overall excluded volume effect. However, when considering the interactions between the glycan beads and the AFM tip, we only used the LJ potential. To suitably parameterise the hat potential we fitted two parameters *F*_max_ and *r*_*c*_ such that the second virial coefficient of the beads was equal to that of the LJ potential with *σ*_LJ_ = 12 nm (see Figs 1 and 2 in the Supplementary Information). Here, the virial coefficient is equal to half of the excluded volume of the bead.

The glycan monomers were connected by harmonic spring and angle bonds. The potential of a harmonic spring bond is given by

5 $${V}_{h}(r)=\frac{J}{2}{(r-{r}_{0})}^{2}$$

where *J* is the spring constant, *r* is the actual distance between bonded particles, and *r*_0_ is the equilibrium bond length. The bond angle potential is similarly defined as

6 $${V}_{a}({\varphi })=\frac{K}{2}{({\varphi }-{{\varphi }}_{0})}^{2},$$

where *ϕ*_0_ = *π* is the equilibrium angle and *K* is the bending stiffness (corresponding to flexural rigidity, *EI*, for rods), which determines the persistence length of the fibres *l*_*p*_ through the relation: *K* = *l*_*p*_ *k*_*B*_ *T*. Here, as expected, *k*_*B*_ is the Boltzmann constant and *T* is the absolute temperature.

In order to model the grafting of the fibres at the surface, we fixed the chains at one end while the remaining monomers were allowed to move. Suitable parameters of the coarse-grained model were set to reproduce the typical spacing, chain contour length, persistence length, and the mean height of the grafted chain over the substrate. The beads were considered to interact with the solvent and with solute monomers by the friction force.

Proteoglycans, attached glycosaminoglycans (GAG) chains, and glycoproteins were considered to be represented by bushes of linear polymers attached to the membrane (or by a brush consisting of individual polymer chains). We assigned the radius values of the beads and the characteristic spacings according to the results of Squire and Weinbaum^18,19^. The bushes were anchored to the substrate on a hexagonal (triangular) lattice with a spacing of 100 nm between the fixed cores (roots). We note that in this work, we are considering only the high-density “healthy” EG. A study of the mechanics of damaged EG with a reduced volume fraction of the layer will be reported in a subsequent paper.

We took a combination of the characteristic length, energy, and dynamic viscosity as a base for our scaling. Their corresponding unit values were equal to $\ell \mathrm{=20}$ nm, ${\mathscr{E}}=1{k}_{B}T=4.14\cdot {10}^{-21}$ J at *T* = 300 K, and *η* = 2 × 10^4^ kg/(m · s). All other units were derived from these basic units. Thereby, one force unit ${\mathscr{F}}={\mathscr{E}}/\ell =2.07\cdot {10}^{-13}$ N, the velocity unit $v= {\mathcal F} /(\eta \ell )$ corresponds to 5.35 × 10^−2^ m/s, and the time unit $\tau =\ell /v=\,3.74\cdot {10}^{-7}$ s. In terms of our scaling, we found that the bending rigidity *K* = 700 pN · nm^2^ (as in ref.^19^) corresponds to a persistence length of approximately 8.14 units (*l*_*p*_ = *K*/*k*_*B*_ *T* = 163 nm), while *K* = 490 pN · nm^2^ (as in ref.^17^) corresponds to 5.4 units. We used these values as a reference and tested whether the simulated EG models reacted to the indentation in a similar way to the experiments for these rigidity values. Other *K* values were also used when studying the role of the filament bending rigidity. We also ran simulations of the EG at *T* = 310 K, which corresponds to body temperature and typical experimental temperatures. While a change from *T* = 310 K to *T* = 300 K may lead to drastic changes in the EG properties *in vivo* and *in vitro*, such a change barely affects the behavior of the model EG. We also compared the EG density distributions at *T* = 310 K and *T* = 300 K and found that the difference between the two does not exceed 3% (see Fig. S9 of the Supplementary Information). We then checked that this change in temperature leads to a negligible difference in the indentation experiments. The measured reaction forces for various models of the EG for indentations of up to 100 nm were found to be the same within the given statistical uncertainty for *T* = 300 K and *T* = 310 K. (see Fig. S10 of the Supplementary Information).

The monomers were initially distributed on a three-dimensional mesh, and the value of *σ*_LJ_ in Eq. (4) corresponds to the sum of the radii of the interacting particles. These bead–bead interactions in the EG were tuned to give an effective bead diameter of *σ*_LJ_ = 0.6 units (12 nm), which equals the sum of two particle radii and their closest approach distance. The distance between the bush cores was chosen to correspond to 5 simulation length units (100 nm), while individual fibres in the SBr and SFBr models were placed 1 simulation unit (20 nm) apart. All bushes were grafted at the membrane at the vertices of a hexagonal (triangular) lattice with a side length of 100 nm. As mentioned above, the monomer chain was such that their contour length corresponded to a length of 500 nm, or 25 simulation units.

In our simulations of the AFM experiments, the spherical tip was slowly immersed into the EG. The tip was modeled as a sphere with a repulsive LJ potential at *r*_off_ = *R* − *σ*_LJ_. We then measured the resulting reaction force by fixing the tip at different positions and calculating the average vertical force exerted on it by the EG beads. At the smaller indentation depths, the time required to achieve the equilibrium values was quite short, but with increasing indentation depth, the equilibration took up to three times longer due to the relaxation of entangled fibres. In all our experimental simulations, the equilibrium was reached and the averaged value curves were extrapolated to ensure there were no residual quench effects. Several series of computational experiments were performed, including different radii of the tip (from 200–500 nm) to check the relation between the tip radius and the reaction force, different EG structures, and for different values of the fibre bending rigidity. The size of the box was chosen to avoid self-interaction between the compressed EG chains and to ensure that there were no periodicity artifacts. Since in several experiments, the radii of the AFM tips used to indent the EG were in the range 1–5 *μ*m^30,32^, to measure the elastic response of the glycocalyx models we used tips with radii *R* = 200, 300, 400, 500 and 1200 nm.

So that we could collect a sufficient amount of data for each level of indentation, the simulation timestep was set to 0.0025 units (≈0.9 ns), and we performed runs of 500,000 to 1,500,000 MD steps. The position of the tip in the *xy*-plane was varied through several configurations in order to get an average value for possible placements of the tip with respect to the bushes (see Fig. S7 of the Supplementary Information). Also, in the simulation of the AFM experiment, we measured the net forces exerted on the bottom constraint and on the upper root particles of the bushes to estimate the pressure transmitted by the EG to the membrane of the endothelial cells. In experimental works, the threshold indentation value for the appearance of the membrane deformation was identified as 100 nm to 200 nm from the luminal surface of the EG. Here, most of our simulations were performed at indentations of up to 150 nm, where we found the membrane deformation to be negligible. Finally, in order to check whether the presence of transverse chains representing hyaluronic acids resulted in a significant difference, we performed a series of simulations with additional cross-linking chains of various lengths (see the Supplementary Information). We used ideal bead-spring chains with zero-sized beads, which were equilibrated inside the EG brush, and for which every tenth monomer was connected by a bond of length 1 to the nearest glycan monomer. We expect such a structure to be closer to models that assume that the brush behaves like a continuous material and which exhibits surface tension. All experiments were visualised using the Visual Molecular Dynamics (VMD) softward package^41^, and Wolfram Mathematica 9 was used to fit our theoretical model to the simulation and experimental data.

## Electronic supplementary material

Supplementary information
