# Supplementary material for: Structure and elasticity of bush and brush-like models of the endothelial glycocalyx
Source: Sci Rep. 2018 Jan 10;8:240. doi: 10.1038/s41598-017-18577-3 (PMC5762753; doi:10.1038/s41598-017-18577-3)
Supplement: Supplementary file 1 — Supplementary information [file 41598_2017_18577_MOESM1_ESM.pdf]

Structure and elasticity of bush and brush-like models of endothelial glycocalyx:

Additional Information

Aleksei Kabedev[1]

Vladimir Lobaskin[1]

[1] University College Dublin, School of Physics, Dublin, 4, Ireland

The model

We used softer pairwise potentials for glycan monomers to allow larger MD time step. To keep the excluded volume effects, we required that the hat potentials between the beads were giving the same second virial coefficients as the Lennard-Jones ones.

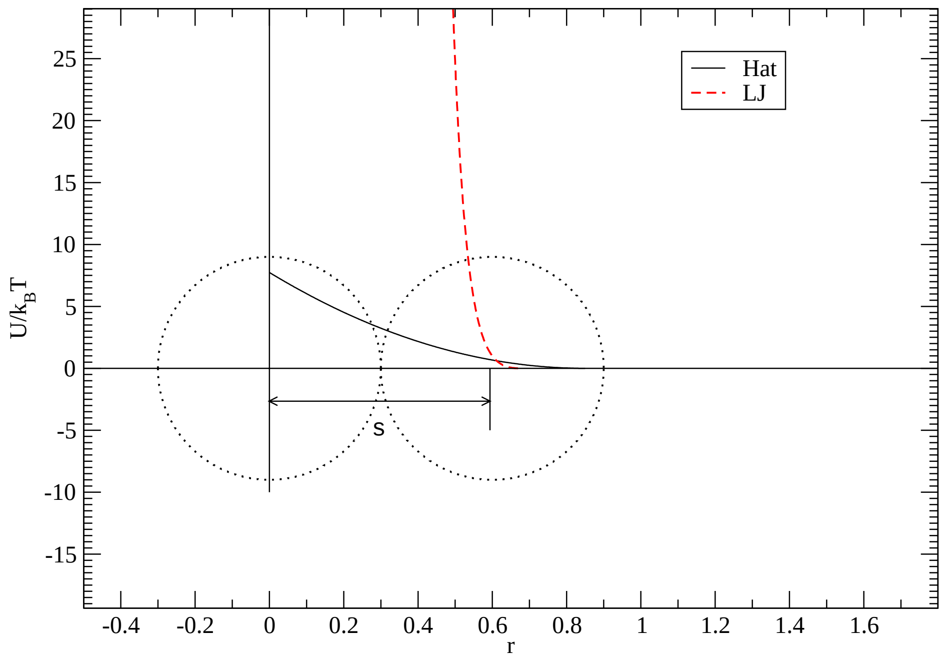

Fig.1: Comparison of Lennard-Jones "12-6" (LJ) and Hat potentials. Hat potential has the same second virial coefficient, but is much softer.

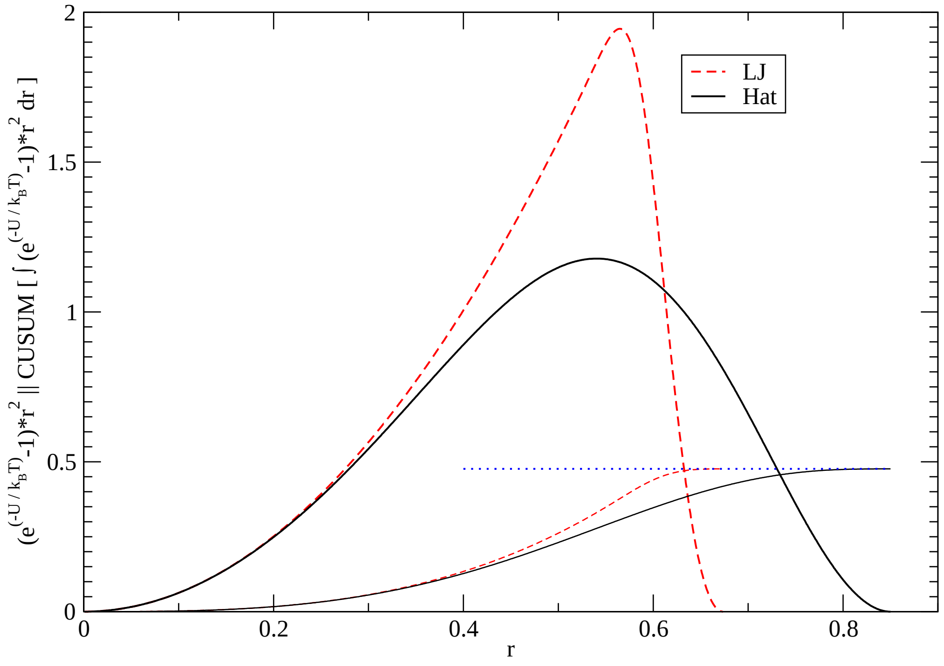

Fig.2: Integrands and cumulative sums of the integrals of the second virial coefficients. For the set of chosen parameters the sums are equal, which results in the same potential of mean force for a foreign particle interacting with the brush.

The density distributions

The graph below (Fig. 3) illustrates the similarities and differences between monomer density distributions in EG with different fiber rigidities and grafting densities. Although in the actual measurements the brushes have different heights, the shape of the upper part of the density distribution, which determines the elastic response at small indentations, is practically the same for all persistence lengths if counted from the point of initial contact. At the same time, the curves for fixed persistence length and different grafting densities show much different slopes. The higher density leads to the higher slope (and larger force on the tip).

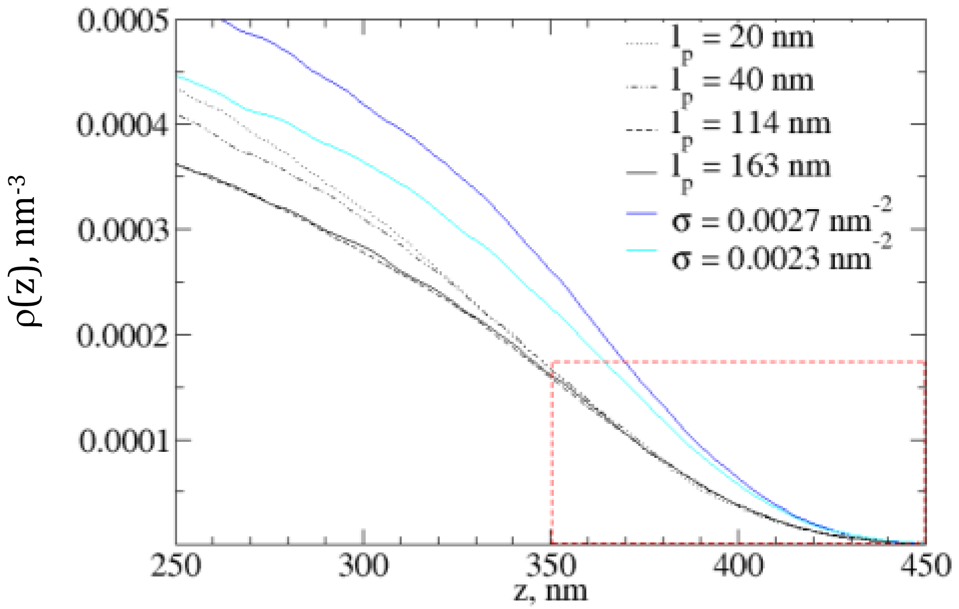

Fig.3: Density distribution functions for different values of persistence length and grafting density are shifted to the same maximum height. The curves with varying persistence length are obtained using SFB at $\sigma = 0.0019$ nm^{-2}, curves for different densities with $l_p =$ 114 nm.

Fig. 4 illustrates the choice of origin (d = 0) for the force-indentation curves. The dots on the curves show to the position of the bottom of the indenter, where the average force on the tip starts to differ from zero. The monomers above that line do not produce any repulsive force on the tip. This position would correspond to initial contact of the AFM tip with EG in experiments.

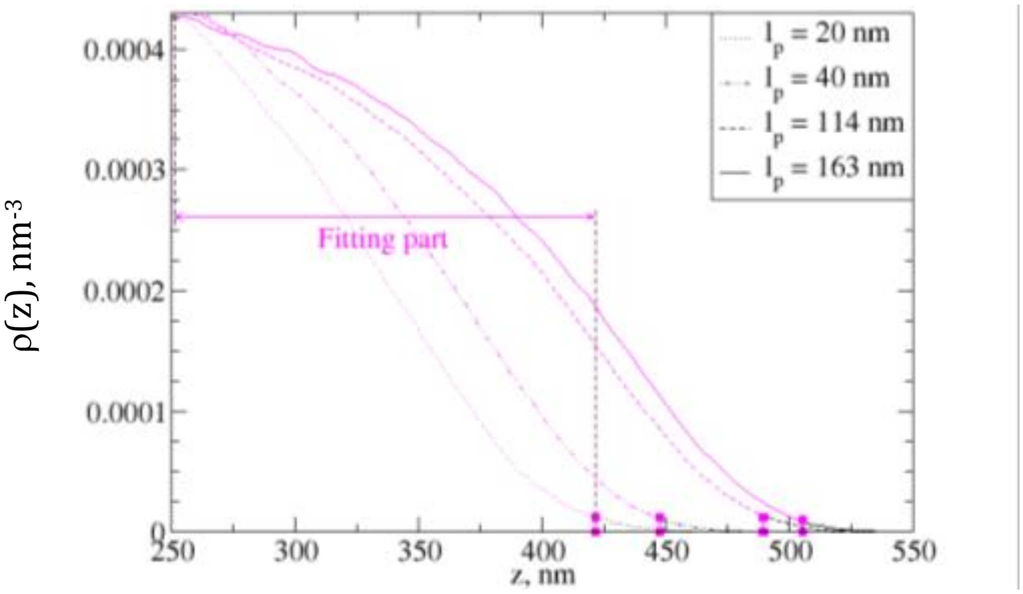

Fig.4: Representation of the part of the grafting density taken for the force curve fitting. The part of the EG beyond the point is not contributing to the force. The curves with varying persistence length are obtained using SFB model at $\sigma = 0.0019$ nm^{-2}.

Fig. 5 illustrates the distribution and conformation of cross-linking chains imitating the hyaluronic acid. We introduced 40 chains of 10 to 60 monomers each (400 nm – 2 μm contour length) with persistent length of 4 nm into the EG in a random fashion, equilibrated them, and then linked every 10th bead of the chain to the nearest glycan monomer by harmonic bonds of the same kind as those used for the brush fibers. The excluded volume of the cross-linking chains was ignored. The corresponding monomer density of the hyaluronans was 0.000625 to 0.00375 nm^{-2}.

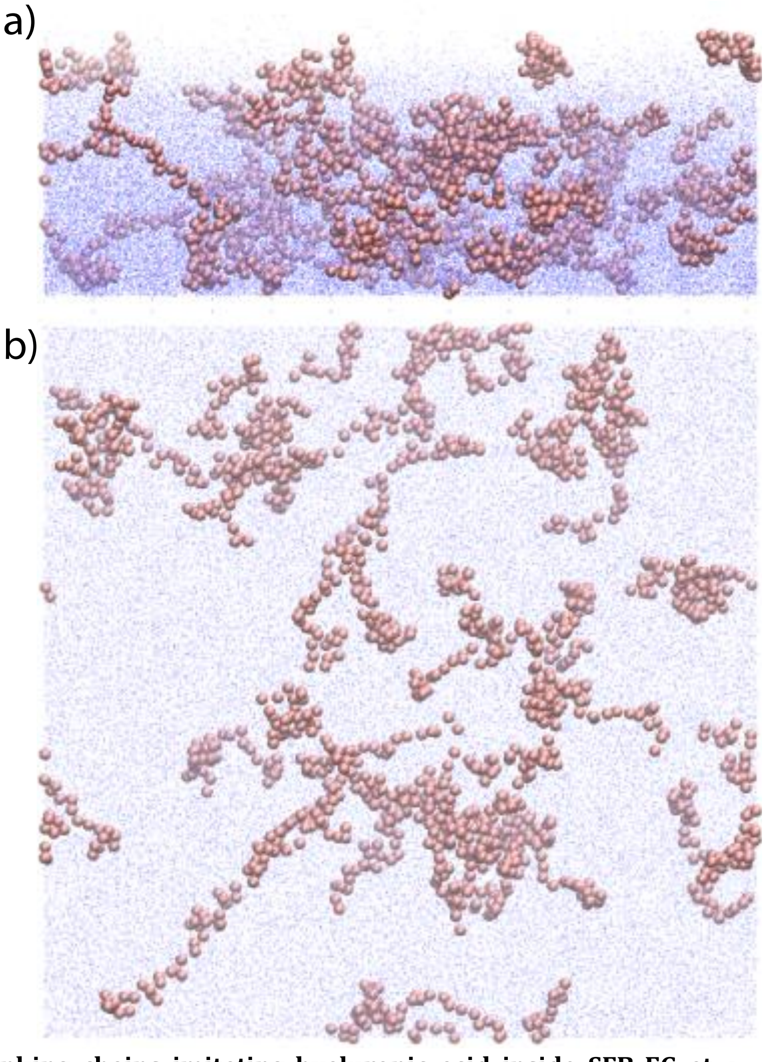

Fig.5: Cross-linking chains imitating hyaluronic acid inside SFB EG at $\sigma = 0.0019$ nm^{-2}. Glycan monomers of the brush are represented by the small blue dots. Side (a) and top (b) views.

The forces

In Fig. 6, we present the measured reaction force from simulation of SFB model EG with and without cross-linking chains. We can see that the presence of the cross-linkers increases the rigidity of the brush but does not change the qualitative picture.

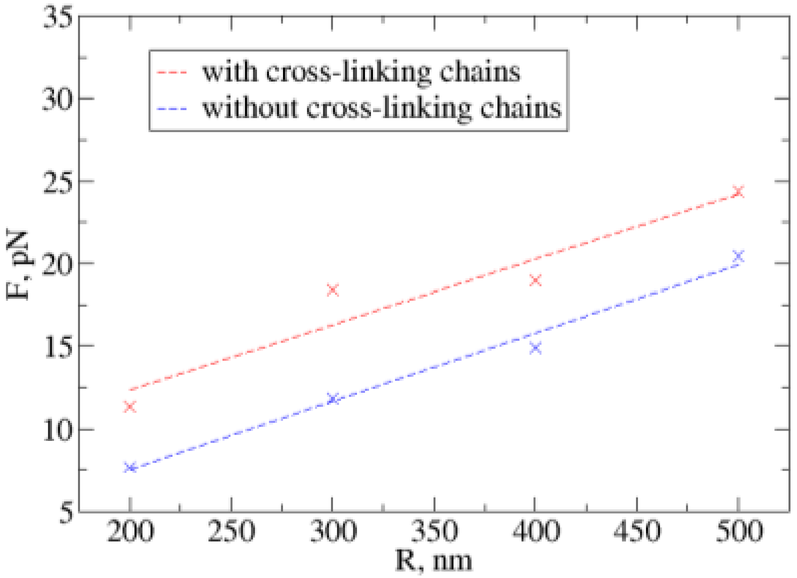

Fig.6: Comparison of the forces at 100-nm indentation on tips of different radii *R* for the EGL containing cross-linking chains and the non-cross-linked one. One can see the linear dependencies on the radius in both cases (the measured rigidity *k* is also the same). Crosses represent the simulation data, dashed lines - linear fitting.

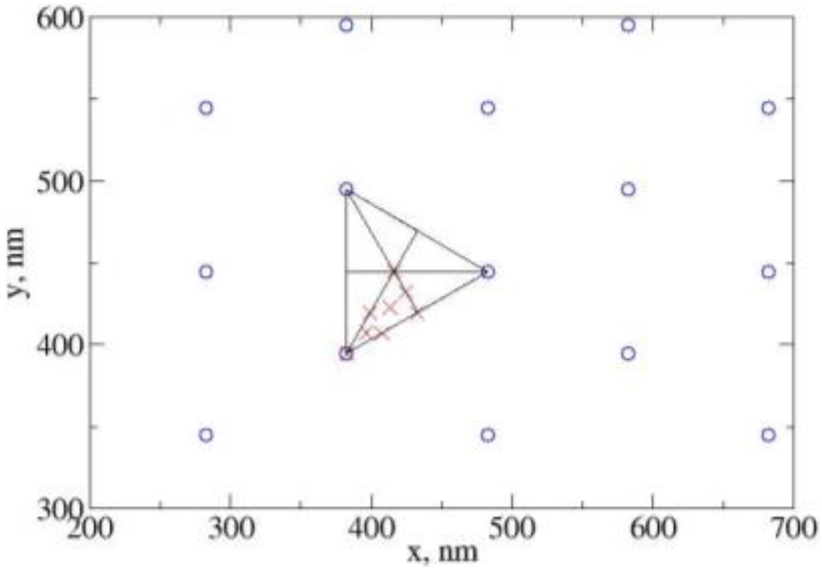

Fig.7: Scheme of the reaction force measurement. The crosses show the sample *XY* positions of the center of the spherical tip with a fixed *Z* position. The location of the bush roots are shown by the blue circles. We than calculated the average over all these positions of the tip (for several configurations of each) to find the force.

Density profile fitting

The equilibrium monomer density profiles in calyces are expressed in nm^{-3} and fitted with function

$$\rho(z) = c_1 z^6 + c_2 z^5 + c_3 z^2 + z_4.$$

Two regions with different coefficients were fitted and the functions matched at the inflection point. An example of the piecewise fit is shown in Fig. 8.

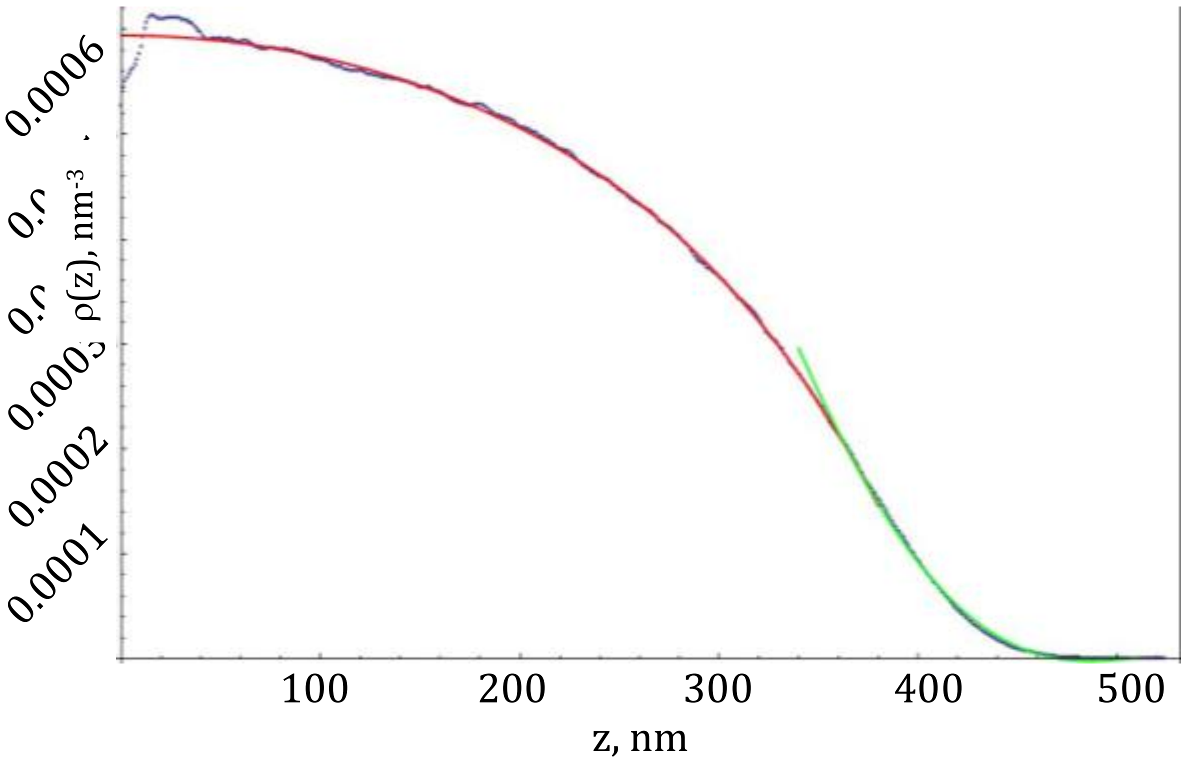

Fig.8: Sample of density distribution function fitting. SFB, l_p=40 nm, σ =0.0019 nm^{-2}: the dotted curve shows the simulation data, the red curve shows the "parabolic" part of the fit, the green curve the sigmoidal part. The pieces are stitched at $z_0 = 365$ nm.

Temperature

The simulations are done using a purely mechanical bead-spring model that does not include any systemic regulation or enzymatic activity at temperature 300 K. To ensure the validity of comparisons with experiment, performed at 310 K, we checked whether a change from 300 K to 310 K would have any effect on the results of simulation. The comparison is presented in Figs. 9 and 10. One can see the glycan monomer density distribution and force profiles for 300 K (T = 1.00 in our simulation units) and 310 K (T=1.03): the differences between the results are less than the statistical uncertainty. Therefore, we used simulation data obtained at 300 K in the paper.

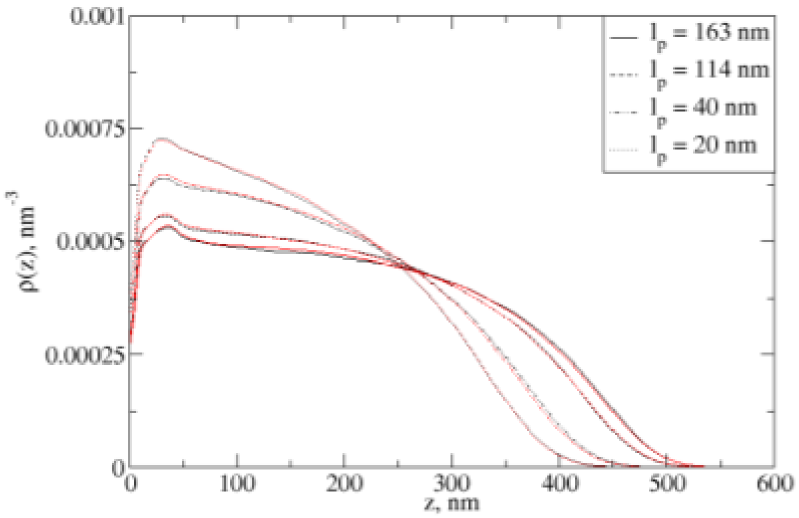

Fig.9: Red lines and black lines stand for T=1.03 and T=1.00 correspondingly

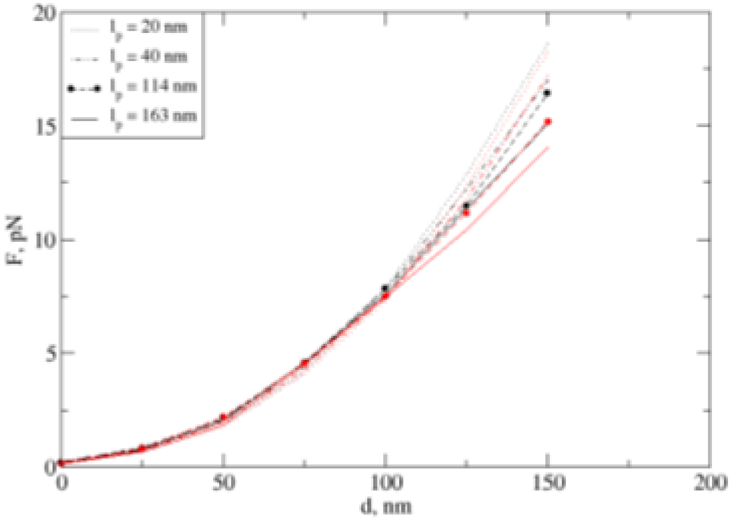

Fig.10: Red lines are for T=1.03 (37°C) and black ones are for T=1 (27°C). As one can see the difference in the range between 0 and 100 nm is negligible.

						Tail part				Parabolic part			
Glycocalyx model	σ, nm^{-2}	l_p, nm	H, nm	z_0 (inflection point), nm	k, pN nm	c_1	c_2	c_3	c_4	c_1	c_2	c_3	c_4
SFB	0.0019	163	505	450	0.67	-2.52E-19	2.20E-16	-2.25E-08	2.68E-03	-1.46E-20	-4.28E-18	-9.52E-10	4.97E-04
SFB	0.0023	163	515	455	0.8	-3.66E-19	3.22E-16	-3.33E-08	4.01E-03	-1.05E-19	4.14E-17	-1.60E-09	5.90E-04
SFB	0.0027	163	520	455	0.87	-3.28E-19	2.95E-16	-3.28E-08	4.13E-03	-1.45E-19	5.98E-17	-1.85E-09	6.79E-04
SFB	0.0019	20	422	355	0.67	-3.23E-19	2.47E-16	-1.67E-08	1.50E-03	3.43E-20	-3.09E-17	-3.23E-09	6.59E-04
SFB	0.0019	40	448	365	0.68	-2.36E-19	1.93E-16	-1.57E-08	1.60E-03	-1.80E-20	-1.19E-17	-2.08E-09	5.94E-04
SFB	0.0019	114	490	420	0.68	-1.72E-19	1.53E-16	-1.62E-08	1.96E-03	-3.10E-20	3.38E-18	-1.35E-09	5.21E-04
FBr	0.0027	163	529	470	0.9	-4.05E-19	3.70E-16	-4.28E-08	5.55E-03	-5.63E-20	1.23E-17	-4.88E-10	6.45E-04
SFBr	0.0027	163	534	470	0.96	-4.86E-19	4.44E-16	-5.14E-08	6.68E-03	-5.74E-21	-1.71E-17	3.81E-10	6.16E-04

Table 1: Table of simulation parameters for various models and equilibrium properties of the corresponding EG brushes. The monomer density profiles are expressed in nm^{-3} and fitted with function $\rho(z) = c_1 z^6 + c_2 z^5 + c_3 z^2 + c_4$.
